# Ultrasound imaging of male urethral stricture disease: a narrative review of the available evidence, focusing on selected prospective studies

**DOI:** 10.1007/s00345-023-04760-x

**Published:** 2024-01-13

**Authors:** Mikołaj Frankiewicz, Malte W. Vetterlein, Karolina Markiet, Jan Adamowicz, Felix Campos-Juanatey, Andrea Cocci, Clemens M. Rosenbaum, Wesley Verla, Marjan Waterloos, Guglielmo Mantica, Marcin Matuszewski

**Affiliations:** 1 Department of Urology, Medical University of Gdańsk, Gdańsk, Poland; 2 Department of Urology, University Medical Center Hamburg-Eppendorf, Hamburg, Germany; 3 Department of Urology, Department of Radiology, University Clinical Centre in Gdańsk, Gdańsk, Poland; 4 Department of Regenerative Medicine, Collegium Medicum, Nicolaus Copernicus University, Bydgoszcz, Poland; 5 Andrology and Reconstructive Urology Unit, School of Medicine, Marqués de Valdecilla University Hospital, Cantabria University, IDIVAL, Santander, Spain; 6 Department of Urology and Andrology, Careggi Hospital, University of Florence, Florence, Italy; 7 Department of Urology, Asklepios Hospital Barmbek, Hamburg, Germany; 8 Department of Urology, Ghent University Hospital, Ghent, Belgium; 9 Department of Urology, AZ Maria Middelares, Ghent, Belgium; 10 IRCCS Ospedale Policlinico San Martino, Genoa, Italy

**Keywords:** Sonourethrography, Urethral ultrasound, Urethral stricture, SUG, Urethra imaging

## Abstract

**Purpose:**

To synthetize the current scientific knowledge on the use of ultrasound of the male urethra for evaluation of urethral stricture disease. This review aims to provide a detailed description of the technical aspects of ultrasonography, and provides some indications on clinical applications of it, based on the evidence available from the selected prospective studies. Advantages and limitations of the technique are also provided.

**Methods:**

A comprehensive literature search was performed using the Medline and Cochrane databases on October 2022. The articles were searched using the keywords “sonourethrography”, “urethral ultrasound”, “urethral stricture” and “SUG”. Only human studies and articles in English were included. Articles were screened by two reviewers (M.F. and K.M.).

**Results:**

Our literature search reporting on the role of sonourethrography in evaluating urethral strictures resulted in selection of 17 studies, all prospective, even if of limited quality due to the small patients’ number (varied from 28 to 113). Nine studies included patients with urethral stricture located in anterior urethra and eight studies included patients regardless of the stricture location. Final analysis was based on selected prospective studies, whose power was limited by the small patients’ groups.

**Conclusion:**

Sonourethrography is a cost-effective and safe technique allowing for a dynamic and three-dimensional urethra assessment. Yet, because of its limited value in detecting posterior urethral strictures, the standard urethrography should remain the basic ‘road-map’ prior to surgery. It is an operator-dependent technique, which can provide detailed information on the length, location, and extent of spongiofibrosis without risks of exposure to ionizing radiation.

## Introduction

Successful treatment of urethral stricture disease requires not only adequate surgical experience but also appropriate preoperative diagnosis. The basic tools widely used for the initial evaluation of patients with suspicion of urethral stricture (US) are uroflowmetry, supplemented with the IPSS (International Prostate Symptom Score) questionnaire. However, these non-invasive tests remain only supplements to the available imaging methods. Currently, the standard imaging of the urethra includes urethroscopy, cystourethrography (CUG) with voiding cystourethrography (VCUG), and increasingly used sonourethrography (SUG) and magnetic resonance urethrography (MRU) [1–3]. Comprehensive data collection is of utmost importance prior to an operation, because factors such as stricture length, location, and extent of periurethral pathology have a key impact on the choice of surgical approach, reconstruction technique, and the final outcome. The implementation of SUG has been already described more than 30 years ago, yet importantly, this method is still evolving. Compared to the first data provided by McAninch in 1988, who was the first to describe implementation of SUG in US diagnosis, the currently widely available high-quality ultrasound devices offer incomparable image quality and detail in the assessment of pathological tissue [4]. The main limitation of the ultrasound technique includes operator dependence and lower sensitivity for evaluation of posterior urethra—a limitation that according to some authors can partly be overcome by the use of transrectal ultrasound [5]. Sonourethrography has shown significant value in several studies and in the light of the growing interest in the application of this method, this narrative review provides a summary of the available literature on the diagnostic role of SUG in the management of urethral strictures. The aim of this review is a thorough analysis of the SUG including technical aspects of the procedure, operator dependency, advantages, and limitations.

### Pathophysiology of urethral stricture disease

The pathophysiology of urethral stenosis is linked to excessive fibrotic growth at the level of the corpus spongiosum. The result of this pathological process is known as “spongiofibrosis”. In contrast to the normal urethral wall, the epithelial layer at the site of stricture is much thicker. Dense packing of elastin fibers around the narrowed urethra causes the loss of natural elasticity of the urethra until they finally prevent proper urination [6]. Fluid’s irritative effect at the site of urethral damage may theoretically intensify the process, but this mechanism has not been practically explored in human studies [7]. It is yet noteworthy, that the first murine model for urinary extravasation revealed that mesenchymal spongiofibrosis can be induced by urethral injury with subsequent extravasation. Understanding of this cause-and-effect sequence explains the need to look for more accurate diagnostic methods that provide information on pathology beyond the urethral lumen [8].

### Conventional urethral imaging techniques: urethrocystography and urethroscopy

Cystourethrography and voiding cystourethrography have been the oldest and most used imaging modalities for patients with US, still being the “gold standard”. The examination is widely accessible and the location and length of the stricture can be evaluated instantly and at a relatively low cost. A great advantage of this method is the ability to assess the entire length of the urethra including the posterior urethra. In the case of complete obliteration of the urethra, in patients who are already on a suprapubic catheter—the proximal segment can be visualized by performing the antegrade urethrogram. Furthermore, CUG/VCUG also detects presence of diverticula, stones, fistula or false path. The main limitation is lack of information about the tissue beyond the lumen of the urethra; thus, information on spongiofibrosis cannot be obtained. Some comparable studies suggest that CUG/VCUG underestimates stricture length [9–14]. Moreover, both the patient and physician may be exposed to ionizing radiation during the procedure, unless an infusion line is used to fill the urethra and bladder. The impact of radiation can be especially significant when repeated examinations are necessary. On the other hand, urethroscopy enables a real-time endoscopic visualization of the urethral lumen without exposure to harmful radiation. Noteworthy, urethrocystoscopy and CUG/VCUG have been considered the preferred tools in post-urethroplasty follow-up protocols to detect a recurrent stricture [15, 16]. Yet, urethroscopy rarely enables assessment of the stricture length as the caliber of symptomatic strictures is usually narrower than the standard cystoscopes used [17]. Moreover, urethroscopy is limited in providing a clear diagnosis in complex cases such as multiple strictures, or complete urethral obliteration.

### Novel urethral imaging technique: magnetic resonance urethrography

Magnetic resonance urethrography stands out among the methods used in the diagnosis of urethral stricture, because it provides three-dimensional images of urethral stricture disease, including data on the tissue surrounding the urethra. One of the major differences, which also determines the choice of one of these methods, is the range of urethra evaluation. Magnetic resonance urethrography was found to be accurate in assessment of both anterior and posterior urethra. The value of MRU is particularly emphasized for the evaluation of the posterior urethra, because preoperative assessment of these strictures correlated more closely with operative findings compared to RUG/VCUG [18–20].

## Materials and methods

**Fig. 1:**
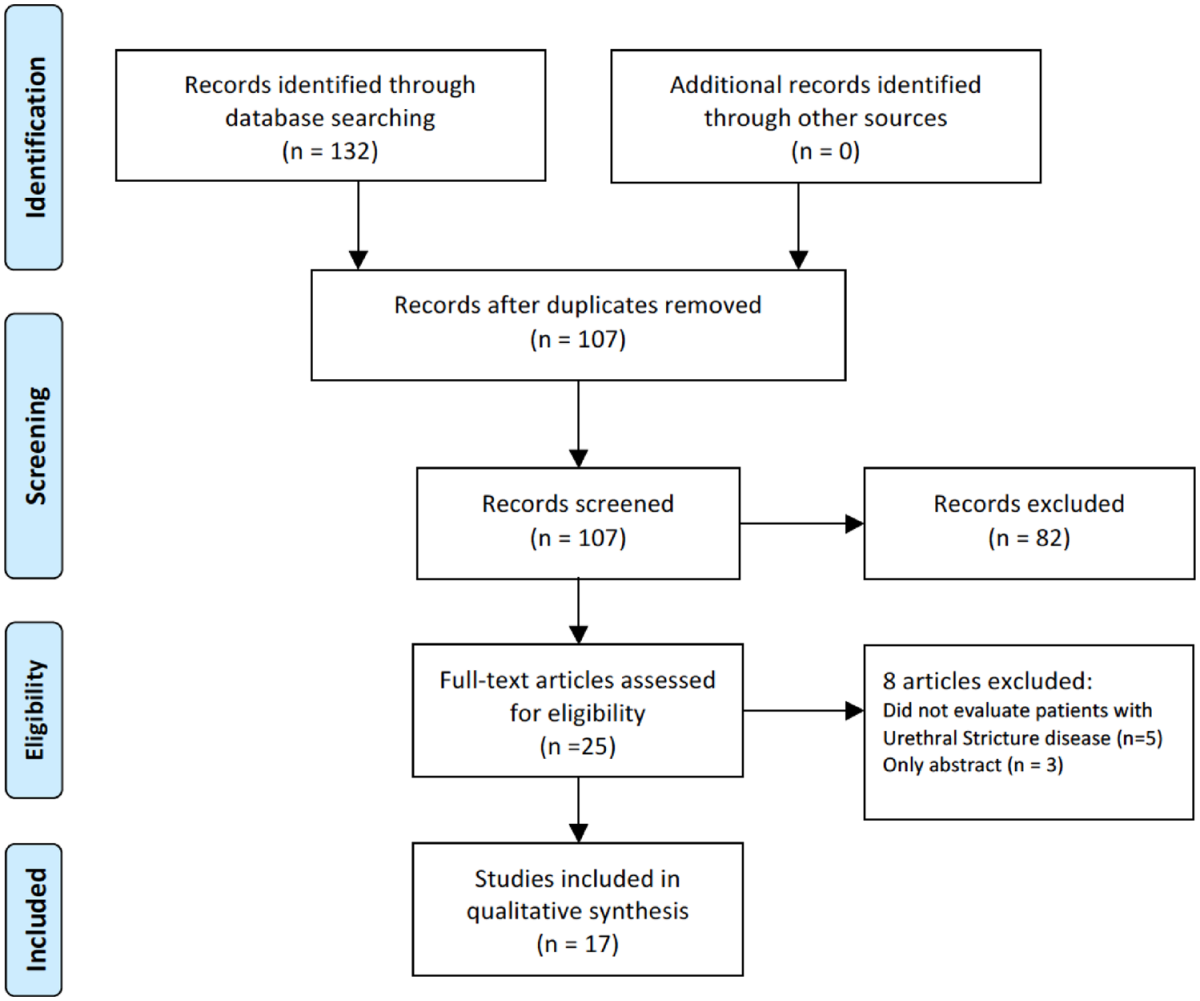
PRISMA flowchart

A comprehensive literature search was performed using the Medline and Cochrane databases in October 2022. Studies that evaluated the use of SUG in the diagnosis of urethral stricture disease were included in the analysis. Prospective studies were selected for this review to obtain the most informative data possible. This exclusion of case reports, editorials, and commentaries, while potentially limiting the scope of the review, was deemed necessary to ensure the highest quality and clinical relevance of the findings. Articles were screened by two reviewers (M.F. and K.M.) who followed the Preferred Reporting Items for Systematic Reviews and Meta-Analysis (PRISMA) statement. The selection process is presented in the PRISMA flowchart (Fig. 1) [21, 22]. The articles were screened using the keywords “sonourethrography”, “urethral ultrasound”, “urethral stricture”, and “SUG”. Only human studies and articles in English were included. Case reports, conference abstracts, editorials, and comments were excluded from detailed analysis.

## Results

Seventeen papers were selected as a result of our literature review on the use of SUG in assessing urethral strictures. Final analysis is based on prospective studies, the majority of which are limited by a small patient population (number of patients varied from 28 to 113). Nine studies included patients with urethral stricture located in anterior urethra and eight studies included patients regardless of the stricture location. As most of the available literature assesses the value of ultrasound based on comparing this method to other modalities and/or surgical findings, the collected data are presented in the Table 1. As shown in the table, the diagnostic accuracy of SUG was compared to RUG/VCUG, MRU, and sonoelastography. The accuracy of SUG was generally high, with most studies reporting a sensitivity of over 80% and a specificity of over 90%. However, there was some variability between studies, with the accuracy of SUG being lower for strictures in the posterior urethra.

**Table 1:** Diagnostic accuracy of sonourethrography compared to other modalities and surgical findings if applicable

Study	Study type	Number of patients	Evaluated segments of the urethra	Compared studies	Intraoperative stricture length measurement	Accuracy of SUG				
						Diagnosis	Location	Length	Diameter	Recurrence
Chung PH et al. 2022	Prospective	28	Bulbar urethra	CEUS SUG, SWE vs. SUG and RUG	Yes	CEUS SUG best correlated with intraoperative stricture length (*R*^2^ 0.709)	–	–	–	CEUS SUG 80% sensitivity, 100% specificity, 93% accuracy
Orakzai ZJ et al. 2022	Prospective	77	Anterior and posterior	SUG vs. RUG	Yes	–	SUG showed better results than RUG	SUG: 62–85% sensitivity, 92–100% specificity, 92–98% accuracy	SUG: 69–82% sensitivity, 95–98% specificity, 83–95% accuracy	–
Frankiewicz et al. 2021	Prospective	55	Anterior and posterior	SUG vs. VCUG, MRU	Yes	–	–	SUG least accurate	–	–
Oyelowo N et al. 2021	Prospective	84	Anterior and posterior	SUG	Yes	–	–	SUG 84.6% sensitivity, 82.7% specificity	–	–
Jesrani A et al. 2020	Prospective	50	Anterior urethra	SUG vs. RUG	Yes	77.5% sensitivity, 96.8% specificity, 90% accuracy	–	–	–	–
Noori D et al. 2020	Prospective	30 with stricture 30 healthy control group	Anterior and posterior	SUG vs. RUG	No	SUG 96.87% accuracy	–	SUG length significantly longer than in RUG (*p* = 0.045)	SUG stricture caliber significantly larger than in RUG (*p* = 0.05)	–
Berna- Mestre et al. 2018	Prospective	113	Anterior and posterior	SUG vs. RUG, VCUG	No	SUG more accurate than RUG (*p* < 0.05)	–	–	–	–
Kalabhavi S et al. 2018	Prospective	30	Anterior urethra	SUG vs. RUG	Yes	–	–	SUG 95% accuracy (*p* < 0.001), RUG 57% (*p* < 0.001)	–	–
Krukowski et al.2018	Prospective	66	Anterior urethra	SUG vs. RUG	Yes	SUG 100% RUG 97%	SUG better correlation in penile urethra *R* = 0.86 (*p* < 0.001), RUG *R* = 0.66 (*p* < 0.001)	SUG better correlation *R* = 0.73 (*p* < 0.001), RUG R = 0.55 (*p* < 0.001)	–	–
Shahsavari R et al. 2017	Prospective	97	Anterior urethra	SUG vs. RUG	No	SUG 86.6% sensitivity 94.6% specificity	–	The length of the stricture in RUG significantly longer than in SUG (*p* = 0.025)	–	–
Bryk D et al. 2016	Prospective	35	Anterior urethra	SUG	Yes	–	–	No significant differences in SUG and intraoperatively (*p* = 0.10) with correlation coefficient of 0.84 (*p* < 0.001)	–	–
Talreja S et al. 2016	Prospective	77	Anterior urethra	SUG vs. RUG and SE	Yes	–	–	SUG 82–92.7% accuracy, RUG 69.5–89% accuracy, SE 87.84–100% accuracy. SE higher accuracy in intermediate and long segment strictures	–	–
Ravikumar et al. 2015	Prospective	40	Anterior and posterior	SUG vs. RUG	Yes	SUG anterior urethra: 100% sensitivity, 100% specificity; posterior urethra 75% sensitivity, 50% specificity RUG 100% sensitivity and specificity	–	More precise estimation with SUG than RUG	–	–
El-Ghar et al. 2011	Prospective	30	Anterior and posterior	SUG vs. RUG vs. MRU	Yes	Anterior urethra: SUG 100% accuracy, RUG 91% sensitivity, 90% specificity, 90% accuracy Posterior urethra: SUG 60% accuracy RUG 89% sensitivity, 91.7% specificity, 90% accuracy MRU 100% sensitivity, 91.7% specificity, 95% accuracy	–	–	–	–
Gupta N et al. 2006	Prospective	52	Anterior urethra	SUG vs. RUG	Yes	–	–	SUG more precise than RUG	–	–
Choudhary S et al. 2004	Prospective	70	Anterior urethra	SUG vs. RUG	Yes	SUG and RUG equally efficacious	–	RUG lower sensitivity (60–80%) for lengths 1–4 cm compared with SUG (73.3–100%)	SUG 50–88% sensitivity, 92–97% specificity, 90–96% accuracy RUG 50–78% sensitivity, 86–96% specificity, 81–94% accuracy	–
Peskar D et al. 2004	Prospective	51	Anterior and posterior	SUG vs. RUG	Yes	SUG identifies 98.4% of RUG strictures	–	No major differences	no major differences	–

CEUS—contrast-enhanced ultrasonography, SUG—sonourethrography, RUG—retrograde urethrography, VCUG—voiding cystourethrography, MRU—magnetic resonance urethrography, SE—sonoelastography, SWE—shear wave elastography

### Sonourethrography technique

The technique of the procedure itself has not changed much since its introduction and most of the authors follow the same steps. The ultrasound transducer should be positioned on the perineal area, and high-frequency ultrasound waves are directed into the urethral tissue. The ultrasound frequencies should be adjusted in different parts of the urethra—15–18 MHz for the penile urethra (from meatus to the distal bulbar urethra) and 9–12 MHz for the bulbar urethra (up to the urethral external sphincter). Special attention should be paid to the impact of the examiner’s pressure created with the transducer against the skin, as too much pressure may generate an impression of a false stricture. Moreover, to avoid artefacts and evaluate the dynamic view of the intraurethral flow, the urethra should be filled during the examination.

The following is a general description of the steps involved in injecting contrast for sonourethrography:

1. Patient preparation: Patient is positioned in a lithotomy position, with legs supported and separated. Perineal area is cleansed with an antiseptic solution.
2. Filling the urethra: A tip of a thin catheter is inserted into the urethral meatus, and the saline is prepared in a syringe. In case of a distal urethra stricture, a blunt plastic cannula can be used. Saline is slowly administered into the urethral lumen, typically in small portions. Any discomfort or adverse reactions should always be noted.
3. Ultrasound imaging: The ultrasound transducer is positioned and moved from the urethral meatus toward the perineal area, and the saline-filled urethra is imaged in real-time. The physician should carefully assess the images for any abnormalities or areas of narrowing. The direction of examination is not relevant; however, the entire length of the urethra available for examination should always be assessed.
4. Post-procedure: Once the imaging is completed, the catheter or cannula is removed, and the patient is instructed to void.

### Anatomy of male urethra on sonourethrography

**Fig. 2:**
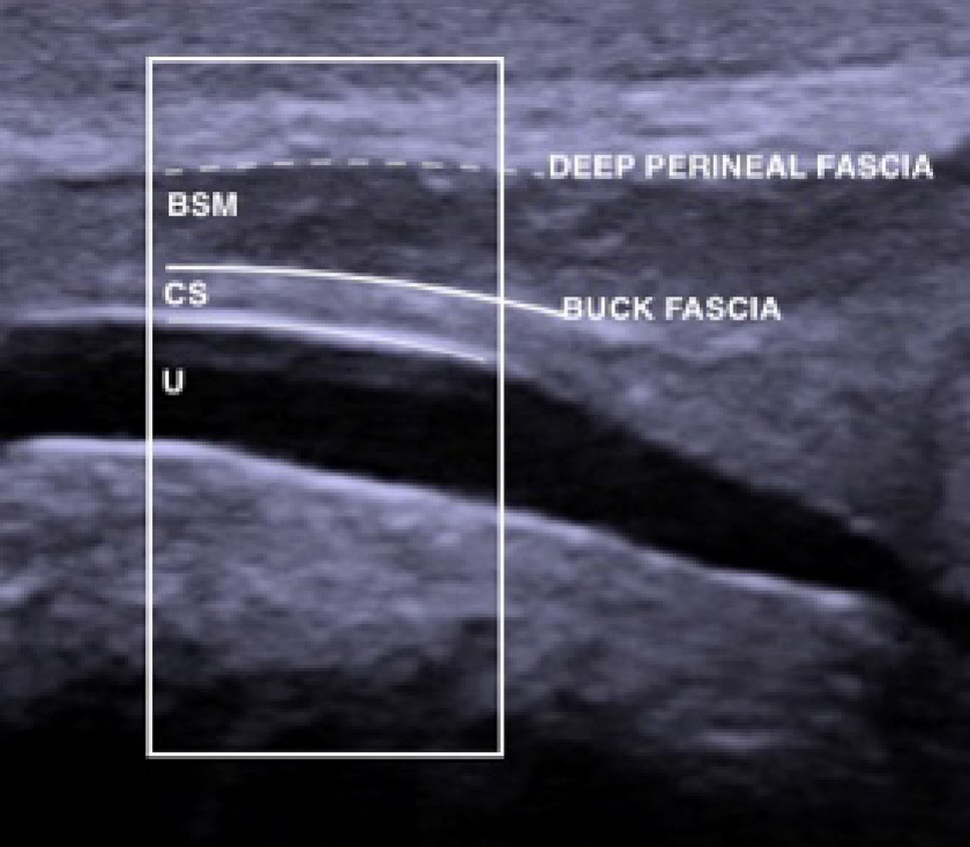
Ultrasound image of bulbar urethra in longitudinal scan shown within the white box (Figure provided by the authors) *U* urethra. *CS* corpus spongiosum. *BSM* bulbospongiosus muscle. Thin white line—urethral epithelium, thick white line —Buck’s fascia, dotted line— *DPF* deep perineal fascia

**Fig. 3:**
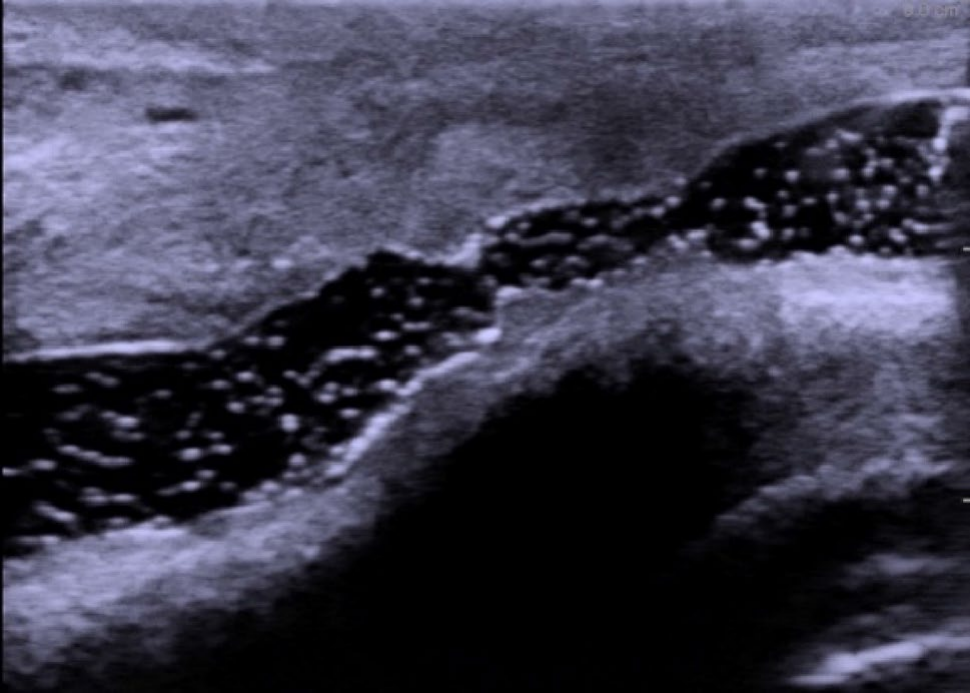
Echoes from saline bubbles are visible within urethral lumen in a patient with stricture. Figure provided by the authors

**Fig. 4:**
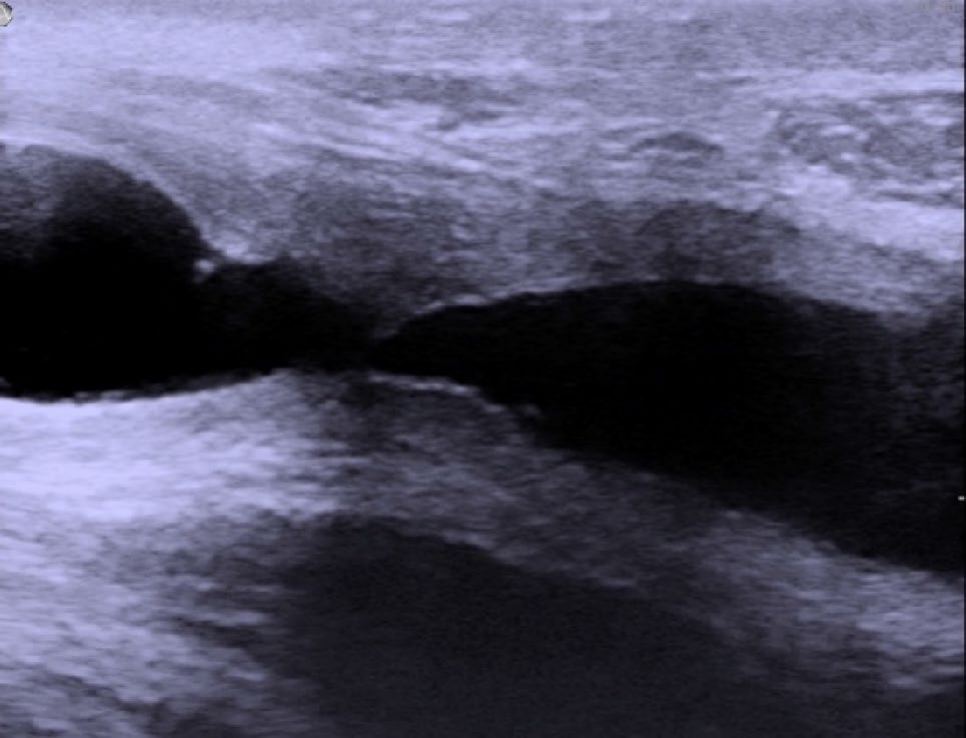
Urethral stricture with spongiofibrosis. Figure provided by the authors

Normal urethra as seen on Fig. 2 presents as an anechoic tubular area, with smooth outline, usually of 8–10 mm in diameter [23]. If saline is introduced, small hyperechoic echoes may be visible within the urethral lumen (Fig. 3). Alterations in course of spongiofibrosis present as hyperechogenic areas in comparison to the normal echogenicity of corpus spongiosum (Fig. 4). Calcifications may be encountered. Ultrasound also allows the evaluation of mucosa and its abnormalities, lumen abnormalities such as diverticula, Cowper glands, paraurethral soft tissues and/or perineal masses, posttraumatic changes, etc., as well as imaging of the bladder (which may show a thickened trabeculated bladder wall in case of high-pressure voiding due to the presence of stricture).

### Additional ultrasound techniques

Sonoelastography also known as virtual or electronic palpation is a novel technique used for measurement of tissue stiffness. Talreja et al., in a study on 77 patients with clinical features of anterior urethral stricture disease concluded that sonoelastography estimates stricture site and length better in comparison with RUG/VCUG and SUG. It estimates the degree of spongiofibrosis which serves as an important prognostic factor for stricture recurrence more accurately than SUG. Despite several subsequent studies, it is not widely used [24–29]. Bosio described contrast-enhanced voiding urosonography (CE-VSUG) via the transperineal approach in a pediatric population after catheter filling of the bladder with ultrasound contrast diluted in serum, and its use for assessing posterior urethral anomalies and the degree of vesicoureteral reflux in children has become widespread [30].

### Sonourethrography vs. other imaging methods

#### Diagnostic accuracy of sonourethrography compared to other methods and surgical findings

Most of the studies compared SUG findings with that of RUG/VCUG in the diagnosis of urethral stricture. In two studies SUG was found to be more accurate at diagnosing stricture presence and estimating the stricture length compared to RUG [9, 10]. Yet, the sensitivity in detecting the stricture and estimating its length using the SUG largely depends on the part of the urethra where the stricture is located. In six studies, SUG has been found to be superior to RUG for anterior urethral strictures [9, 10, 26, 31–33]

The highest correlation for stricture length at operation was for strictures located in the penile urethra [6]. Another early study comparing SUG to conventional RUG found that RUG tended to underestimate actual stricture length as compared to SUG [32, 34]. Tembhekar and colleagues evaluated the role of SUG in 70 male patients referred to the urology department for symptoms suggestive of urethral stricture disease. This study diagnosed 39 strictures in 33 patients. RUG/VCUG and SUG were equally efficacious in diagnosing anterior urethral strictures; however, only one of three (33.3%) posterior urethral strictures were adequately visualized on SUG. The group also concluded that SUG was superior in evaluating spongiofibrosis; however, this appeared to be subjective, based on authors’ opinion. Interestingly, 61 of the 70 (87%) of patients involved in this study preferred SUG over conventional RUG, as it was felt to be less invasive and caused less discomfort [35, 36]. Only in one study, SUG was the least accurate method compared with RUG/VCUG and MRU with average overestimation of 2 mm as related to the operative measure [18]. Despite high accuracy of SUG in most patients, the authors of this study experienced some notable outliers in the SUG measurements. None of these problems occurred in the penile urethra; instead, they were all exclusive to the bulbar or membranous urethra. This accurately depicts the technical challenges of performing SUG in the posterior urethra, which is nearly impossible despite optimal patient placement and considerable operator expertise [18, 37]. Also, it was discovered that in 44 out of 232 (19%) patients undergoing anterior urethral reconstruction included in the study, the results of the intraoperative SUG changed the planned reconstructive technique (based on the preoperative RUG). The authors of this study described criteria to perform an anastomotic urethroplasty based on the intraoperative urethral ultrasonogram findings demonstrating a bulbar urethral stricture length of < 25 mm on aggressive urethral distension [38].

#### Sonourethrography for the assessment of spongiofibrosis

Most authors concluded that SUG enables the evaluation of spongiofibrosis in the anterior urethra and provides similar accuracy as compared to MRU. More anatomical detail is MRU’s principal benefit, which is offset by the cost of the modality and the difficulty of image interpretation. A qualitative and quantitative evaluation of spongiofibrosis may also be provided by SUG incorporating real-time elastography [26, 30, 37]. It is yet unknown whether determining the exact extent of spongiofibrosis before the surgery has significant clinical value and is still to be investigated in further research. However, most authors agree that it has an influence on the choice of surgical technique as excision of the fibrotic fragment and end-to-end anastomosis is preferred in the case of extensive spongiofibrosis [38]. In a study by Ravikumur et al. [31], SUG appeared to more accurately depict stricture length, stricture diameter, and degree of spongiofibrosis when correlated with cystoscopic and intraoperative findings.

#### Sonourethrography as a sole imaging technique

Most of the articles that have been published demonstrate the value of SUG as an auxiliary modality in addition to the standard methods of diagnosing urethral strictures such as RUG or urethroscopy. However, in a recent study, Bryk and colleagues evaluated the viability of using SUG as the sole imaging technique for diagnosing urethral strictures prior to surgical treatment. This study demonstrates that, in a high-volume center with an experienced team, SUG may be the sole imaging modality needed to plan a definitive urethral reconstruction. It should be highlighted that this study only included patients with anterior urethral strictures. In comparison to RUG, which was 90% accurate in this study of 30 men who underwent both procedures, SUG was 100% correct for anterior urethral strictures, but only 60% accurate for posterior urethral strictures. Hence, as the authors concluded, it is not recommended to extend these findings to the posterior urethra. In the light of available data on SUG, because of its limited value in detecting posterior urethral strictures, the standard urethrography should remain the basic ‘road-map’ prior to surgery, particularly in patients with suspected urethral stricture undergoing initial diagnosis [39].

## Highlights and clinical indications

Retrograde urethrography has historically been the gold standard for identifying urethral strictures; however, because of its certain drawbacks, novel imaging techniques have been investigated and evaluated. Before deciding on surgical intervention, it is crucial to thoroughly consider the length, location, number of the strictures and their morphology since each may affect the choice of the treatment method. Modern high-resolution ultrasound is widely available; thus, the quality of data provided by this diagnostic method has improved significantly since the first description several decades ago. Sonourethrography has nowadays become a viable supplement to the standard modalities and provides additional valuable information. Fibrous scarring of the corpus spongiosum leading to a decrease in the urethral lumen is the fundamental theory explaining the pathogenesis of urethral stricture disease. Sonourethrography provides data on spongiofibrosis with satisfactory accuracy making this method widely used mostly in specialized reconstructive urology centers. As a high-resolution, multi-planar, and cost-effective technique that can be performed in an outpatient setting, SUG has found its place in the new standards of diagnostics of anterior urethral strictures. It is safe for both the patient and the physician because neither are exposed to radiation. Moreover, the possibility of using saline instead of iodine contrast makes it applicable also for allergic patients.

However, knowing in which clinical situations SUG is of the greatest value is crucial. As proven in numerous publications, the satisfactory accuracy of the SUG refers primarily to the penile urethra. Some authors question the value of the radiological assessment of strictures of the distal urethra and its impact on the choice of surgical technique. These strictures are often extensive or multiple, rather than single as mostly observed in iatrogenic bulbar strictures. Thus, regardless of length and extent of spongiofibrosis, these strictures often require onlay urethroplasty with opening the urethral lumen when most accurate assessment of the pathology may be achieved during the surgery. On the other hand, in these cases, SUG seems to be the best method to show the periurethral pathology up to the urethral opening with high accuracy, allows discussion of the surgical plan with the patient before the surgery. Moreover, SUG can be of particular use to calculate the flap width in the pendulous urethra, where fasciocutaneous flaps are frequently used for reconstruction. For this purpose, Morey and McAninch proposed a straightforward formula 26–3 D (where D is the urethral diameter in mm) [40]. The lumen diameter can be measured with satisfactory accuracy with ultrasonography. This prevents excessive flap width from causing urine pooling and enables the fasciocutaneous flap to be harvested before the urethra is opened.

Furthermore, SUG can be particularly valuable in cases when conventional ascending urethrography is challenging or impossible due to the anomalous anatomy of the distal urethra. This is particularly the case in patients with hypospadias, when both the native and reconstructed urethra are often extremely difficult to evaluate. While descending SUG avoids the need to inject a contrast agent, micturating SUG, although challenging, is feasible even in very complex cases and does not require catheterization of the urethra. The use of SUG in these patients should also be particularly considered as a follow-up tool—without exposing the patient to radiation. Thus, future research should investigate the accuracy of sonourethrography in the follow-up of patients after urethral stricture surgery. This could be a way to detect early recurrence of the stricture. In addition, research is ongoing to develop new ultrasound techniques that can improve the accuracy and clinical utility of sonourethrography. For example, researchers are exploring the use of three-dimensional ultrasound and contrast-enhanced ultrasound.

One of the significant limitations of SUG is operator dependency and although the statistical analysis on the issue is scarce or non-existent, nearly all papers stress that despite wide availability of ultrasound and inclusion of the technique in both urethral stricture diagnostic algorithms and guidelines, it has not yet been entirely incorporated into urological everyday practice [41–43]. Also the issues of long learning curve, limitation in evaluating the posterior urethra, technical aspects of the examination, such as patient preparation and the length of the examination itself are being raised [43, 44].

## Conclusion

Sonourethrography assessment of the male anterior urethra in patients with anterior urethral strictures is a safe, well-tolerated, minimally invasive and cost-effective diagnostic modality. For the posterior urethra, this technique cannot be recommended, based on the available published evidence. While more studies are needed to better characterize SUG, it could be proposed as an additional diagnostic modality, especially in severe and recurrent cases. More evidence on SUG and more data from studies with larger patients' groups need to be collected in the next future, as so far no randomized clinical trials have been published. Although in the future, SUG might replace CUG/VCUG as the investigation of choice in the diagnosis of anterior urethra strictures, at present, combining RUG/VCUG still remains the gold standard in evaluating urethral stricture disease.

## Data Availability

This article is a narrative review that synthesizes findings from existing literature. It does not contain any new data generated by the authors. Data supporting the findings of this review are available within the cited articles. Readers are referred to these original publications for access to the specific datasets analyzed.
